# Supplementary material for: Evidence of Antibiotic Resistance and Virulence Factors in Environmental Isolates of Vibrio Species
Source: Antibiotics (Basel). 2023 Jun 16;12(6):1062. doi: 10.3390/antibiotics12061062 (PMC10295579; doi:10.3390/antibiotics12061062)
Supplement: Supplementary file 1 [file antibiotics-12-01062-s001.zip › antibiotics-2402365-supplementary.pdf]

Table S1: Results of biochemical test assay of environment water samples isolates

Sample code	Biochemical assessment results of environmental water sample isolates											
	Vog. Pro.	Arg Ut	Slt Tol	ONPG	Ctr Ut	Orn Ut	Mnt	Arb	Su	Glu	Sln	Clb
V-Gan	+	+	+	+	+	+	-	-	-	-	+	-
VH-I3	+	+	+	+	-	-	-	-	-	-	-	+
VH-I4	+	+	+	+	+	+	-	-	-	-	+	-
VH-II1	-	-	+	+	+	-	-	-	-	-	-	-
VH-II2	-	-	+	+	-	-	-	+	+	+	+	+
VH-II3	-	-	+	-	+	-	-	-	-	+	-	-
VH-II4	+	+	+	+	+	+	-	-	-	-	+	-
VHMC-A	+	+	+	+	+	+	-	-	-	+	-	-
VHMC-B	-	-	+	+	+	+	+	+	+	+	+	+
VHMC-C	+	+	+	+	+	+	+	+	+	+	+	+
VHMC-D	+	+	+	+	+	+	-	-	-	+	-	+
VHVB-I	+	+	+	+	+	+	-	-	-	-	-	+
VHVB-II	-	-	+	+	+	-	+	+	-	+	+	+
VR	+	+	+	+	+	+	-	-	-	-	-	+
VM-IA	-	+	+	+	+	+	-	-	-	+	-	-
VM-IB	-	+	+	+	+	+	-	-	-	-	-	+
VM-IIA	+	+	+	+	+	+	-	-	-	+	-	-
VM-IIB	-	-	+	+	+	+	+	-	+	+	-	+
VP-IA	+	+	+	+	+	+	-	-	-	+	-	-
VP-IB	+	+	+	+	+	+	-	-	-	-	-	+

Vog Pros = Voges Proskauers, Arg Utl = Arginine Utilization, Slt Tlr = Salt tolerance ONPG = Ortho Nitro Phenyl beta Galactosidase, Ctr Utl = Citrate Utilization, Orn utl = Ornithine Utilization, Mnt = Mannitol, Arb = Arabinose, Su = Sucrose, Glu = Glucose, Sln = Salicin, Clb = Cellobiose

Table S2: Antibiotic resistance and susceptibility assessment of each isolate

S/N	Isolates	Diameter of zone of inhibition (in mm)													
		AMP	GEN	FZD	NA	CHL	TET	CEX	CTX	CPFX	CMX	NOR	STR	NEO	PMB
**1**	***E. coli* DH5α**	15	20	14	20	21	19	15	26	24	24	24	15	15	28
2	**VHVB-I**	RES	24	15	25	25	28	31	05	28	RES	25	23	ND	ND
3	**VHVB-II**	RES	17	11	26	25	18	15	34	25	16	22	16	ND	ND
**4**	**VH-I4**	RES	19	15	30	16	20	06	29	26	19	25	15	ND	ND
**5**	**VH-I3**	RES	28	14	RES	26	20	RES	22	20	16	20	20	21	22
**6**	**V-Gan**	07	15	11	ND	19	17	RES	19	20	19	18	RES	15	15
**7**	**VH-II1**	RES	20	10	ND	27	20	08	34	25	17	24	19	19	12
**8**	**VH-II2**	RES	20	13	ND	26	28	RES	20	24	15	24	12	20	17
**9**	**VH-II3**	06	20	06	ND	30	21	08	15	27	18	17	19	20	08
**10**	**VH-II4**	08	16	10	ND	22	18	ND	ND	ND	ND	ND	ND	15	12
**11**	**VHMC-A**	16	24	20	28	ND	19	20	34	32	30	30	20	20	16
**12**	**VHMC-B**	RES	30	18	RES	ND	20	RES	32	26	RES	20	20	22	22
**13**	**VHMC-C**	RES	18	14	18	ND	21	20	26	24	RES	20	16	RES	14
**14**	**VHMC-D**	RES	20	RES	RES	ND	16	RES	RES	20	RES	22	14	20	16
**15**	**VR**	RES	14	12	16	22	28	12	25	20	13	19	12	16	12
**16**	**VM-IA**	10	19	17	RES	19	22	12	22	17	RES	14	10	19	11
**17**	**VM-IB**	RES	19	15	19	23	24	RES	20	25	19	20	15	17	13
**18**	**VM-IIA**	09	15	12	RES	22	18	RES	21	23	20	22	15	13	15
**19**	**VM-IIB**	RES	19	13	RES	29	20	15	26	17	RES	15	16	16	10
**20**	**VP-IA**	11	12	13	20	20	26	RES	20	20	19	20	12	12	14
**21**	**VP-IB**	RES	15	09	13	22	21	RES	13	25	RES	21	15	12	10

ND: Not determined RES: Resistant

AMP: Ampicillin, GEN: Gentamycin, FZD: Furazolidone, NA: Nalidixic acid, CHL: Chloramphenicol, TET: Tetracycline, CEX: Cephalexin, CTX: Cefotaxime, CPFX: Ciprofloxacin, CMX: Co-trimoxazole, NOR: Norfloxacin, STR: Streptomycin, NEO: Neomycin, PMB: Polymyxin B

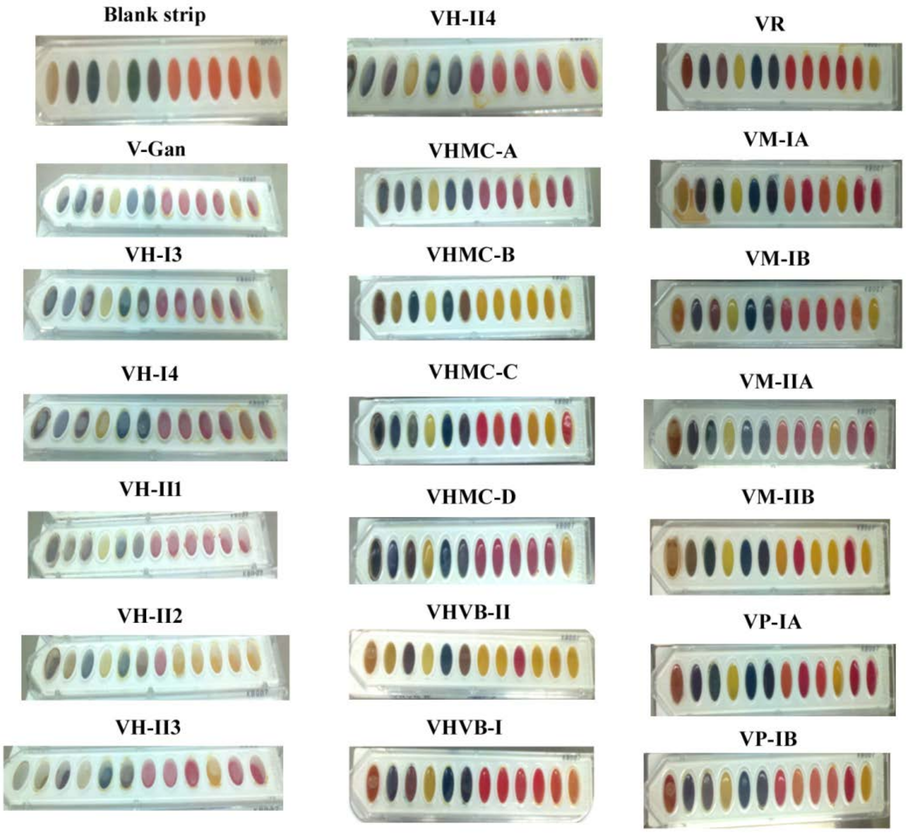

Figure S1: Result of biochemical test confirmation of each *Vibrio* isolates
